# A SURVEY OF FALLS AND ASSOCIATED FACTORS AMONG OLDER COMMUNITY-DWELLERS

**DOI:** 10.1093/geroni/igz038.3158

**Published:** 2019-11-08

**Authors:** Meng-Ling WU, Shang-Yu Wu, Chien-Ning Tseng

**Affiliations:** 1 Oriental Institute of Technology, New Taipei, Taiwan; 2 Guoxian Chinese Medicine Clinic, New Taipei City, Taiwan (R.O.C.), Taiwan; 3 Department of Nursing, Oriental Institute of Technology, Nil, Taiwan

## Abstract

After surveying elderly people in northern Taiwan with a structured questionnaire to collect basic information and experience of falls in the past year. In total, 250 valid questionnaires were gathered. The SPSS software package used in statistical analysis of data and a p-value <0.05 was considered to reflect statistical significance by Chi-square (χ2) test. In this study, 176 female accounted for 70.4%; 213 people living with family accounted for 85.2%; 68 people aged 66-70 years old accounted for 27.2%; 85 people with public education at the elementary level accounted for 34%. 156 people with falling experience accounted for 62.3%; 60 people who fell home accounted for 38.5%; 74 people who fell during outdoor activities accounted for 47.4%; 63 people fell in walking, cycling and sports, accounting for 85.1%. In addition, there is a significant correlation between residents’ medication and fall experience; Such as antihypertensive drugs, hypoglycemic agents, and analgesics, are more statistically significant about fall; and taking sedative, muscle relaxation, Chinese medicine, there is no statistically significant. This shows that it is very important to prevent the fall of the elderly and the safety of the elderly during outdoor activities. In addition to the need to increase the muscles and strength of the elderly, it is also necessary to strengthen the outdoor space and drug education for the elderly.

